# Military deployment correlates with smaller prefrontal gray matter volume and psychological symptoms in a subclinical population

**DOI:** 10.1038/tp.2016.288

**Published:** 2017-02-14

**Authors:** O Butler, J Adolf, T Gleich, G Willmund, P Zimmermann, U Lindenberger, J Gallinat, S Kühn

**Affiliations:** 1 Max Planck Institute for Human Development, Center for Lifespan Psychology, Berlin, Germany; 2 Charité University Medicine, Campus Charité Mitte, Clinic for Psychiatry and Psychotherapy, Berlin, Germany; 3 Psychotrauma Center of the German Military, Military Hospital Berlin, Berlin, Germany; 4 European University Institute, Department of Political and Social Sciences, Badia Fiesolana, San Domenico di Fiesole, Italy; 5 Max Planck UCL Centre for Computational Psychiatry and Ageing Research, Berlin, Germany; 6 University Clinic Hamburg-Eppendorf, Clinic and Policlinic for Psychiatry and Psychotherapy, Hamburg, Germany

## Abstract

Research investigating the effects of trauma exposure on brain structure and function in adults has mainly focused on post-traumatic stress disorder (PTSD), whereas trauma-exposed individuals without a clinical diagnoses often serve as controls. However, this assumes a dichotomy between clinical and subclinical populations that may not be supported at the neural level. In the current study we investigate whether the effects of repeated or long-term stress exposure on brain structure in a subclinical sample are similar to previous PTSD neuroimaging findings. We assessed 27 combat trauma-exposed individuals by means of whole-brain voxel-based morphometry on 3 T magnetic resonance imaging scans and identified a negative association between duration of military deployment and gray matter volumes in ventromedial prefrontal cortex (vmPFC) and dorsal anterior cingulate cortex (ACC). We also found a negative relationship between deployment-related gray matter volumes and psychological symptoms, but not between military deployment and psychological symptoms. To our knowledge, this is the first whole-brain analysis showing that longer military deployment is associated with smaller regional brain volumes in combat-exposed individuals without PTSD. Notably, the observed gray matter associations resemble those previously identified in PTSD populations, and concern regions involved in emotional regulation and fear extinction. These findings question the current dichotomy between clinical and subclinical populations in PTSD neuroimaging research. Instead, neural correlates of both stress exposure and PTSD symptomatology may be more meaningfully investigated at a continuous level.

## Introduction

Stress is a pervasive element of modern life, and the detrimental effects of stress on physical and mental health^1, 2, 3^ have long been recognized. On a neural level, the neurotoxic effects of stress are known to contribute to gray matter alterations in animals.^2, 4^ Chronic stress, or the administration of glucocorticoids, has been shown to result in volumetric reductions in hippocampal and prefrontal regions including the anterior cingulate cortex (ACC) and ventromedial prefrontal cortex (vmPFC) through reductions in neurogenesis and dendritic atrophy.^5, 6, 7^ More recently, neuroimaging methods have allowed *in vivo* investigation of the effects of stress on the structure and function of the brain in humans.

Neuroimaging research into the effects of stress in humans has focused mainly on post-traumatic stress disorder (PTSD). PTSD is a debilitating psychiatric disease characterized by intrusive memories, hyperarousal, emotional numbing and avoidance.^8^ Research on PTSD has reported similar findings to those on stress in animal studies, including smaller gray matter volumes in hippocampal and prefrontal brain regions.^9, 10, 11, 12, 13^ A key role for these regions in PTSD symptomatology is supported by their well-established role in memory function,^14^ executive control processes,^15^ emotion regulation and fear extinction.^16, 17^ However, it is an open question in the PTSD neuroimaging literature whether locally reduced gray matter volume represents a pre-existing risk factor, or is acquired, either following stress exposure or with the onset of symptomatology. First evidence from twin studies suggests that reduced hippocampal volumes represent a risk factor for PTSD,^18^ whereas prefrontal reductions are acquired.^19^

Trauma exposure is a necessary condition for the development of PTSD.^20^ However, the effects of trauma exposure on brain structure, in individuals who do not meet a clinical threshold, remain largely unexplored. Neuroimaging research on subclinical individuals is needed to clarify whether previously observed gray matter differences reflect a dichotomy between patient populations and trauma-exposed controls, or rather a spectrum of stress-related brain changes. The latter seems more likely, given evidence of a dose–response relationship between stress exposure and PTSD, with previous traumas, severity of trauma and additional life stress all posing significant risk factors for PTSD.^21^ In functional neuroimaging research, results suggest that trauma exposure can have enduring effects on the brain, even in individuals without PTSD.^22^

Military deployment is one specific instantiation of exposure to repeated stress and trauma. In recent years, military and political conflict across the globe has increased, resulting in greater numbers of individuals experiencing combat and continuous exposure to extreme stress. In addition to acute stressors, there is growing recognition of non-specific deployment related ‘wear and tear' as a source of stress injury.^23^ Military deployment has many elements that may contribute to wear and tear, including separation from family and loved ones, disturbances to sleeping patterns, changes in diet, and limited opportunity for rest. In addition, deployed military personnel are also exposed to traumatic events, loss or grief, potential moral conflict between their ethical beliefs and the reality of their combat experiences, or a combination of these factors.

Neuroimaging research in this field has focused almost exclusively on clinical populations. However, only a fraction of individuals will actually develop PTSD during or after military deployment, with prevalence of post-deployment PTSD varying by severity of exposure and population characteristics.^24, 25, 26, 27^ In previous work assessing rates of PTSD in German soldiers, 85% of soldiers deployed overseas suffered at least one traumatic event. However, the incidence of first-time PTSD in the 12 months following return from deployment was only 0.9%,^27^ whereas overall rates of PTSD in German troops range between 0.6 and 2.9%.^26, 27^ With rates of around 300 cases of PTSD for every 10 000 returning German soldiers,^27^ the current sample of combat trauma-exposed individuals without psychopathology represent the majority of deployed individuals within the German Armed Forces. By focusing on a non-clinical population that has been exposed to trauma, the current study addresses an important gap in neuroimaging research into stress and PTSD.

In this study, we thus examined brain-structural correlates of military deployment in a non-clinical population using magnetic resonance imaging (MRI). We chose duration of military deployment in days as an objective and verifiable measure of stress exposure that is directly comparable across individuals. We consider this to be more robust than a measure such as combat experiences, which relies on recollection months or years after an event has occurred, and which fails to capture the non-specific effects of deployment related ‘wear and tear'. We also consider military deployment in days to be a more stable measure of stress exposure than PTSD questionnaire scores, which assess symptoms experienced over the last week or month. Trauma is known to produce lasting effects on the brain.^28^ Therefore, the more stable the measure of stress exposure, the more likely it will capture the long lasting effects of stress on brain. We examined whether smaller gray matter volumes previously observed in PTSD populations are also present in individuals exposed to combat trauma who did not receive a psychiatric diagnosis. We also explored associations between military deployment, gray matter volume and PTSD symptoms. We hypothesize that as stress exposure—as indexed by duration of military deployment—increases, gray matter volume in regions previously implicated in PTSD—namely the vmPFC, ACC and hippocampus—decreases. In addition, based on previous research demonstrating a mediating role of gray matter between stress exposure and psychological distress,^3, 18, 29, 30, 31^ we hypothesize that alterations in gray matter will correlate more strongly than military deployment with PTSD symptoms.

## Materials and methods

### Participants

Twenty-eight male soldiers with mission-related trauma but without mental illness were recruited from the German Federal Armed Forces. All participants had been deployed overseas to areas of conflict and were screened by clinical psychologists for the presence of mission-related trauma within the last 2 years (inclusion criteria) and for current or previous Axis I psychiatric disorders (exclusion criteria) according to ICD-10 criteria^20^ using the Mini-DIPS.^32^ The Mini-DIPS is a well-established structured interview for point and lifetime prevalence of AXIS I disorders and has been shown to be valid and reliable for the diagnosis of mental disorders.^32^ The local Ethics Committee of Charité University Clinic, Berlin, Germany, approved of the study, and written informed consent was obtained from each participant prior to participation. One participant was identified as an age outlier (*Z*-score>±2.5) and excluded from subsequent analysis. The mean age of the remaining 27 participants was 32.33 years (s.d. 5.3, ranging between 23 and 42). Participants had completed on average 3.4 military deployments (s.d.=2.6, ranging between 1 and 11), with an average total of 363 days of military deployment (s.d.=246, ranging between 22 and 900) in their military career. This sample size allowed us to detect large effects (*r*⩾0.5), at *α*=0.05 and 80% power^33^ as can be expected based on a previous meta-analysis of PTSD neuroimaging studies.^12^

### Scanning procedure

Structural images were collected on a Siemens Tim Trio 3T scanner (Erlangen, Germany) using a standard 12-channel head coil. The structural images were obtained using a three-dimensional T1-weighted magnetization prepared gradient-echo sequence (MPRAGE) based on the ADNI protocol (www.adni-info.org; repetition time=2500 ms; echo time=4.77 ms; TI=1100 ms, acquisition matrix=256 × 256 × 176, flip angle=7; 1 × 1 × 1 mm^3^ voxel size).

### Questionnaires

Prior to neuroimaging all participants completed a number of questionnaires assessing psychological symptoms and experiences during deployment. Participants completed German versions of the following self-report questionnaires (see below for details); the Post-traumatic Diagnostic Scale (PDS), the Brief Symptom Inventory (BSI), the Interpretation of PTSD Symptoms Inventory (IPSI) and the Posttraumatic Cognitions Inventory (PTCI), as well as a study-specific questionnaire that included items on the number and duration of military deployments.

The PDS^34^ is designed to aid in the diagnosis of PTSD according to the DSM IV criteria and assess symptom severity. As part of the PDS respondents rated 17 items representing the main symptoms of PTSD experienced in the past 30 days, on a four-point scale.

The BSI^35^ is a 53-item self-report questionnaire measuring nine symptom dimensions of psychological distress (for example, somatization, depression, anxiety and hostility). Each item was rated on a five-point scale ranging from 0 (not at all) to 4 (extremely), based on the intensity of distress over the past week. For the BSI, we utilize the Global Severity Index (GSI) score, as this is recommended as the single best indicator of current psychological distress levels.^35^ The GSI is calculated by taking the mean of the nine subscales.

The IPSI^36^ assesses appraisal of PTSD symptomatology over the past month, rated from 1 (totally disagree) to 7 (totally agree).

The PTCI^37^ is a 33-item questionnaire on negative post-trauma appraisals over the past month, relating to the self, the world, and self-blame, rated from 1 (totally disagree) to 7 (totally agree).

A summary score across all four symptom-oriented questionnaires was also calculated. This is appropriate from a theoretical perspective because all four questionnaires are designed to assess trauma-related symptoms and psychological distress, and also justified empirically, given that the four questionnaire scores correlated positively among each other.

### Data analysis

Structural data were processed with voxel-based morphometry (VBM8, http://dbm.neuro.uni-jena.de/vbm.html) and statistical parametric mapping (SPM8, http://www.fil.ion.ucl.ac.uk/spm) using default parameters running on MATLAB 8.1 (Mathworks, Sherborn, MA, USA). VBM is a neuroimaging analytic technique that allows whole-brain investigation of focal differences in brain anatomy based on statistical parameter mapping of structural images. It involves bias correction, tissue classification, and affine registration. Images were normalized to Montreal Neurological Institute (MNI) space and segmented into gray matter, white matter and cerebrospinal fluid based on voxel signal intensity and *a priori* expectation of tissue type based on anatomical location, using default parameters. Modulation was applied in order to preserve the volume of a particular tissue within a voxel by multiplying voxel values in the segmented images by the Jacobian determinants derived from the spatial normalization step. In effect, the analysis of modulated data tests for regional differences in the absolute amount (volume) of gray matter. Images were smoothed with a full width at half maximum (FWHM) kernel of 8 mm.

A whole-brain voxel-wise multiple regression with days of deployment was computed. Age and total gray matter volume were entered as covariates of no interest. Total gray matter volume was used as a covariate of no interest to control for global differences in gray matter and to increase the specificity of regional effects.^38^ The resulting maps were thresholded at *P*<0.001 and cluster-extent thresholded with family-wise error (FWE) correction at *P*<0.05 in combination with correction for non-isotropic smoothness^39^ to control for type-I error.

The Region-of-Interest Extraction (REX) Toolbox^40^ was used to extract gray matter volumes from the resultant clusters, creating one value per participant per cluster.

To explore the relationship between military deployment and related gray matter volumes to psychological symptoms, zero-order bivariate correlations between deployment-related gray matter volume, days of deployment, age and the mean score of the four symptom-oriented questionnaires were estimated. OpenMx 2.2.4 [ref. 41] under R 3.2.1 [ref. 42] was used to obtain maximum likelihood point estimates of the correlations and, importantly, 95% likelihood-based confidence interval estimates. Likelihood-based intervals perform relatively well in samples of small size and are sensible given bounded parameter spaces as is the case with correlations.^43^ Indeed, a quick simulation with 10 000 replications for a sample size of 27 cases and a correlation matrix similar to the one estimated here, reveals very good coverage rates of above 94% per parameter. To ease optimization, the data were *z*-standardized prior to analysis, correcting for the extreme intervariable differences in scale and location.

## Results

Participants reported an average symptom severity score on the PDS of 4.26 (s.d.=3.99, ranging between 0 and 16). A symptom intensity score between 1 and 10 is considered mild, whereas a score of between 11 and 20 is considered moderate. None of the participants met all the criteria for PTSD according to the PDS, further supporting the clinical assessment of these individuals as without psychopathology. For the BSI, we utilize the GSI score.^35^ A T score of 63 or above on the GSI is considered clinically significant, whereas the mean for our current population was 38.15 (s.d.=10.95, ranging between 24 and 70). For the IPSI a clinically significant score for individuals with PTSD is 3.4 (±1), whereas the mean score among the participants was 1.9 (*s.d.*=0.55, ranging between 1.36 and 3.54). For the PTCI we used the total score, which is the sum of the individual scores for the 33 statements. In the original paper by Foa *et al.*, the score for individuals with PTSD was 133 (±44), while the score for traumatized individuals without PTSD was 49.^37^ The mean of our current sample, 48.07 (s.d.=15.07, ranging between 33 and 90), reflects our recruitment criteria.

**Figure 1:**
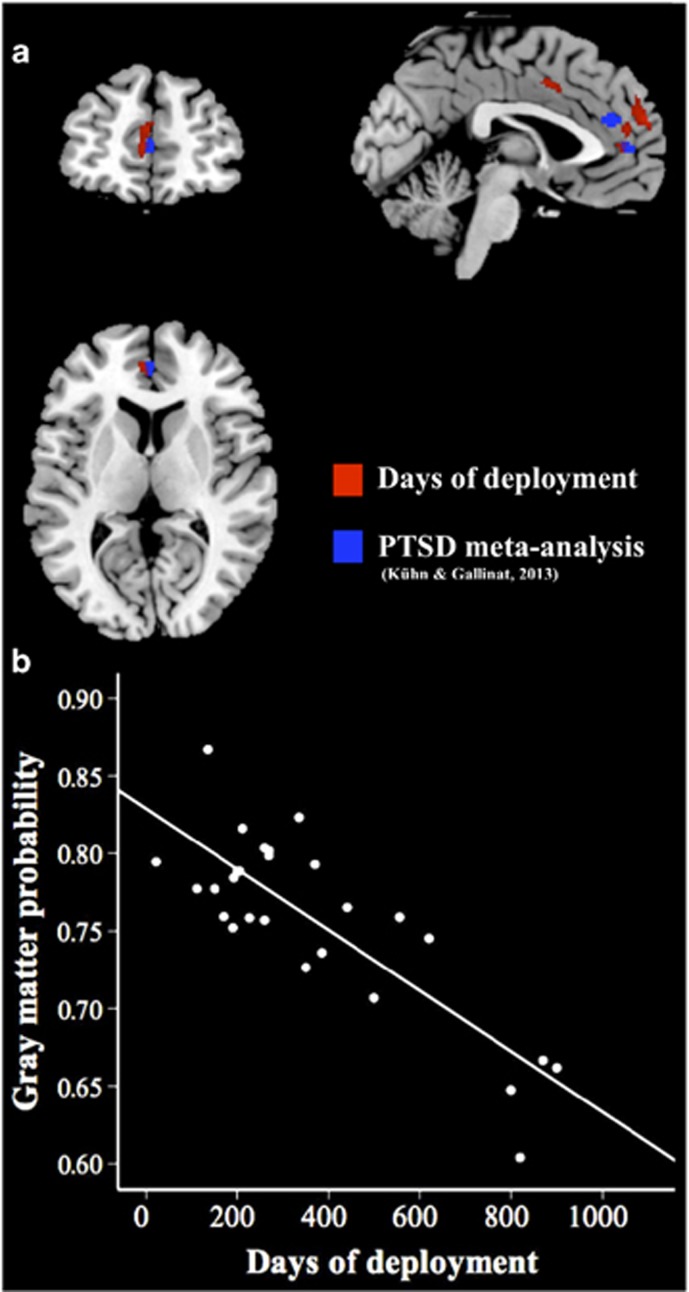
(**a**) Brain regions showing a significant negative correlation between military deployment and gray matter volume in left ventromedial prefrontal cortex (vmPFC; MNI coordinates: *x*=−9, *y*=63, *z*=23; *P*<0.05, *k*=405, family-wise error and non-stationary smoothness corrected) and in bilateral dorsal anterior cingulate cortex (ACC; MNI coordinates: *x*=3, *y*=8, *z*=44; *P*<0.05, *k*=290) are shown in red. Structural reductions from a meta-analysis comparing patients with post-traumatic stress disorder (PTSD)^13^ with controls are shown in blue. (**b**) The scatterplot depicts the correlation between the combined gray matter probability values of the two regions per individual and days of deployment. MNI, Montreal Neurological Institute.

Whole-brain regression analysis yielded a significant negative association between days of deployment and gray matter volume in left vmPFC (MNI coordinate peak voxel *x*=−9, *y*=63, *z*=22.5; *P*<0.05, *k*=405, FWE cluster-extent threshold corrected) and in bilateral dorsal ACC (*x*=3, *y*=7.5, *z*=43.5; *P*<0.05, *k*=290; Figure 1a). No region showed a significant positive association between gray matter and days of deployment. For visualization purposes, a scatterplot shows the combined gray matter volume for both regions plotted against days of deployment (Figure 1b).

In order to test the robustness of the result, leave-one-out cross validation analysis was conducted. The analysis was re-run 27 times with 26 participants, excluding a different participant each time. The resulting maps were thresholded at *P*<0.001 and cluster-extent thresholded with FWE correction at *P*<0.05 in combination with correction for non-isotropic smoothness. A one-sample *t*-test was conducted on the corrected maps from the negative correlation between days of deployment and gray matter volume. The resulting clusters were also located in the left vmPFC (MNI coordinate peak voxel *x*=−1.5, *y*=48, *z*=7.5, *P*<0.05, *k*=361, FWE cluster-extent threshold corrected) and bilateral dorsal ACC (*x*=−1.5, *y*=6, *z*=43.5, *P*<0.05, *k*=175, FWE cluster-extent threshold corrected). No positive correlation between days of deployment and gray matter volume survived in any of the analyses.

We failed to observe a predicted correlation between hippocampal volume and days deployment, even at a more lenient significance threshold of *P*_(uncorrected)_<0.001.

**Figure 2:**
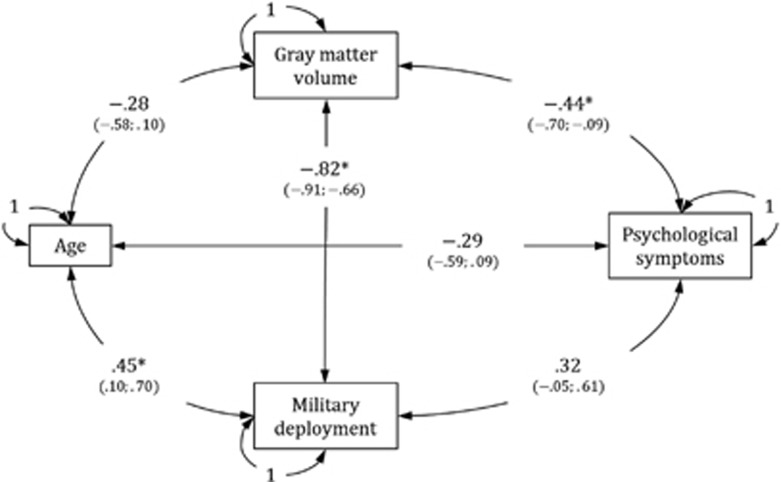
Correlations between psychological symptoms, deployment-related gray matter volume from clusters identified in the ventromedial prefrontal cortex (vmPFC) and anterior cingulate cortex (ACC), days of deployment, and age. Maximum likelihood point estimates are reported along with 95 percent likelihood-based confidence interval estimates in brackets. *P<0.05; note that a 95% confidence interval estimate excluding zero implies that the corresponding effect is significantly different from zero at a significance level of 0.05.

The correlations between the psychological symptom score (that is, the summary score on the four symptom-oriented questionnaires), deployment-related gray matter volume from the clusters identified in the vmPFC and ACC, days of deployment, and age are shown in Figure 2.

The psychological symptoms score was significantly negatively associated with gray matter volume (that is, the corresponding confidence interval excludes zero) but did not show a reliable association with days of deployment. Although in the latter case one might speak of a trend towards a positive effect, as the lower interval boundary was close to zero. The correlation between deployment-related gray matter volume and days of deployment reflects the results also described in the whole brain regression analysis. Note that the confidence intervals were generally wide (that is, the correlation point estimates are relatively imprecise), reflecting the small size of the sample. No effect was found for age.

## Discussion

In the present study, we investigated the neural and behavioral correlates of repeated stress exposure in trauma-exposed soldiers. Individuals with longer histories of military deployment showed smaller volumes in the left vmPFC and bilateral dorsal ACC. The regions observed in the current study overlap directly with those previously observed to be smaller in a PTSD meta-analysis, where trauma-exposed healthy controls were compared with PTSD patients.^13^ The current findings provide support for the detrimental effects of stress exposure on gray matter volume, also in subclinical populations and have two main implications. First, they point towards a reduction of prefrontal gray matter in response to environmental stressors. This may indicate that at least some of the smaller gray matter volumes observed in PTSD represent a consequence of, rather than a risk factor for, stress exposure and stress related psychopathology. However, longitudinal research is required to establish the directionality and nature of these effects. Secondly, as stress exposure in subclinical individuals increases, a pattern of smaller prefrontal gray matter emerges, similar that observed in PTSD patients. This has important implications for future neuroimaging studies of stress exposure and PTSD. The current dichotomy between individuals with and without a clinical diagnosis may not be supported at the neural level, and instead neural correlates of both stress exposure and symptomatology may be more meaningfully investigated at a continuous level.

In functional MRI studies the vmPFC and dorsal ACC have been implicated in affective and cognitive processing, respectively, including the regulation of fear expression, memory and emotional processing.^44^ Structural reductions in these two related but functionally distinct regions could explain the dual psychopathologies observed in PTSD: affective symptoms such as hyperarousal and numbing^8^ as well as cognitive symptoms including memory deficits^45, 46^ and failure to learn fear extinction.^14, 17^

Specifically, the vmPFC is known to have a key role in self-referential processing and the extinction of conditioned fear. A neurocircuitry model of PTSD^47^ proposes that there is a failure to inhibit a fear reaction in response to threat, through reduced top–down control by the vmPFC and reduced bottom–up control by the hippocampus on the amygdala.^48^ In an earlier study by this group,^49^ fMRI was used to assess blood flow while participants were shown images of happy and fearful faces. Patients with PTSD showed increased amygdala and reduced medial PFC responsivity to fearful versus happy facial expressions, compared with controls. In addition, PTSD symptom severity was negatively correlated to activity in the medial PFC during this task.^49^

On the other hand, the ACC is involved in multiple processes, including modulation of affect, memory, response to pain, autonomic and endocrine functions and visceral responses.^50, 51^ The dorsal ACC is a proposed cognitive subdivision of the ACC, forming part of the attention network and involved in the modulation of attention, motivation, error detection and working memory,^52^ as well as modulating fear expression.^53^ Gray matter reductions in the dorsal ACC have previously been observed in PTSD populations exposed to both single-event trauma (due to a terrorist attack)^54^ and chronic (abuse-related) trauma.^55^

We did not observe a correlation between hippocampal volume and days of deployment. Some evidence suggests that smaller hippocampal volumes may only be present in individuals with severe or chronic PTSD. In some studies smaller hippocampal volumes in PTSD patients versus controls were only observed when individuals with less severe PTSD were excluded from the analysis,^18^ while other studies including patients with less severe PTSD failed to find smaller hippocampal volumes.^56, 57^ Another possible explanation for the divergence of findings may be that a smaller hippocampal volume may constitute a risk factor for PTSD. In a study of monozygotic twins discordant for combat exposure, Vietnam veterans with PTSD and their non-combat exposed co-twins had smaller hippocampal volumes than those of combat exposed non-PTSD veterans and their co-twins.^18^ This finding suggests that smaller hippocampi are not the result of traumatization but rather constitute a risk factor for PTSD. PTSD symptom severity of the exposed twin was also negatively correlated with hippocampal volumes of the non-exposed twin. This was taken as evidence that smaller hippocampal volumes may precede stress exposure and increase risk for psychopathology. In contrast, a later study on the same data suggested that reduced gray matter in regions including the ACC represent stress-induced loss, with veterans with PTSD showing smaller volumes than their non-combat exposed co-twins or combat veterans without PTSD.^19^

In the current study, we observed correlates of military deployment in a subclinical population that mirror previous findings in populations with diagnosed PTSD, including research that compared individuals with PTSD to combat trauma-exposed controls (that is, participants similar to those examined here). In contrast to our study, most combat PTSD neuroimaging research examines data from participants with a long interval between combat exposure and assessment. In a meta-analysis of PTSD studies reporting smaller gray matter volume in PTSD, five of the six combat-related studies included reported an interval of over ten years between trauma and assessment.^11^ The remaining study, which did not report the duration of the interval, failed to show evidence of hippocampal atrophy.^58^ In the current study there was a relatively short period between combat-related stress exposure and assessment (less than two years). It may be that over longer periods, healthy individuals recover gray matter, while individuals with psychopathology do not.

On the basis of prior knowledge about detrimental effects of stress on brain structure^7^ and predictive links of smaller gray matter volumes to PTSD,^18^ symptom severity,^29^ and response to therapy,^30^ we hypothesized that combat exposure would lead to increased activation of the stress response, both during traumatic events, and through anticipation and expectation of such events. This increased stress activation would be expected to result in altered brain chemistry and structure, leading to greater susceptibility to the psychological impact of events and subsequent psychological distress. In line with this assumption, we found that smaller deployment-related gray matter volumes in the vmPFC and ACC correlated with higher scores on the psychological symptom-oriented questionnaires. In addition, military deployment itself did not significantly correlate with psychological symptoms, emphasizing the utility of neuroimaging data over and above questionnaire data.

It needs to be kept in mind, however, that the reported associations between brain structure, military deployment, and psychological symptoms are cross-sectional in nature and represent between-person differences whose etiology has not been observed. Longitudinal data are needed to learn more about the temporal dynamics and possibly reciprocal causal effects among these variables. This would also help in studying potential links between pre-existing between-person differences and between-person differences in stress events, physiological reactivity, and distress. Of particular interest would be the question whether the effect of stress exposure per se on stress symptomatology is mediated by stress-related brain changes, and whether this mediation is modulated by pre-existing individual differences.

The potential mediating role of brain structure in the development of psychological symptoms also has implications for intervention. It has previously been shown that stress-related brain changes are reversible and can be altered by both pharmacological and psychological therapeutic interventions, as well as lifestyle interventions such as diet changes, exercise, rest, and stress avoidance.^31^ Interventions aimed at recovering lost gray matter may protect against subsequent development of psychopathology. In addition, increases in gray matter may also correlate with increased response to therapy. Studies of PTSD patients scanned prior to therapy showed that larger gray matter volumes predicted better response to therapy and increased symptom reduction.^29, 30^ In addition, alterations in gene expression and increases in hippocampal volume have been observed in patients with PTSD following therapy, with increases in hippocampal volume predicting reductions in PTSD symptomatology.^59^

We recognize that the duration of military deployment is a broad index of stress exposure. However, we propose that it is nevertheless a conceptually useful variable that ties into current theories of stress injury, including wear and tear,^23^ and allostatic load.^60, 61^ Duration of military deployment is also an objective and verifiable measure that does not rely on subjective interpretation months or years after an event has occurred, and is directly comparable across individuals.

## Conclusion

To summarize, the present findings reveal associations between military deployment and gray matter volume among personnel without a clinical diagnosis of PTSD that are similar to those also observed in PTSD populations. To our knowledge, this is the first structural MRI study using VBM to provide evidence for an association between military deployment and gray matter in individuals with no current or previous psychopathology. The present findings, in the context of previous findings in patients diagnosed with PTSD, question the current dichotomy in the neuroimaging literature between clinical and subclinical populations. Indeed, trauma-exposed subclinical individuals may provide a useful and hitherto overlooked population when exploring the effects of stress on the brain, made all the more relevant by the fact that these individuals represent the majority of trauma exposed individuals. These results also underscore the need for future work to include objective and continuous measures of trauma exposure.

One potential limitation is the specific nature of the participant group and the generalizability of the current findings. Other adult populations with chronic stress exposure and high rates of PTSD, such as first responders, may also show similar changes in prefrontal gray matter, even in subclinical populations. Indeed, these results may also extend to single traumas, such as motor vehicle accidents or personal assault, and to individuals with high levels of perceived stress in their daily life. However, neural effects may be more pronounced in military populations as trauma sequelae are likely to be exacerbated during deployment by multiple exposures within a short period of time, and limited opportunity for rest. Future work is needed to explore how the current results extend to non-military and female samples.

In addition, the data point to a relationship between psychological symptoms, gray matter volumes in ACC and vmPFC, and military deployment. Further longitudinal research is necessary to disentangle the causal chain of effects, that is, whether increased stress exposure leads to altered brain structure, greater susceptibility to the psychological impact of events, and subsequent psychological distress.
